# Supplementary material for: Pancreatic islet chromatin accessibility and conformation reveals distal enhancer networks of type 2 diabetes risk
Source: Nat Commun. 2019 May 7;10:2078. doi: 10.1038/s41467-019-09975-4 (PMC6505525; doi:10.1038/s41467-019-09975-4)
Supplement: Supplementary file 2 — Description of Additional Supplementary Files [file 41467_2019_9975_MOESM2_ESM.pdf]

Description of Additional Supplementary Files

Supplementary Data 1: Accessible chromatin sites in pancreatic islets

Supplementary Data 2: Sequence motifs enriched in islet accessible chromatin sites

Supplementary Data 3: Hi-C loops identified in pancreatic islets samples

Supplementary Data 4: Gene promoters in direct chromatin loops with islet enhancer elements

Supplementary Data 5: Islet enhancers in direct chromatin loops to gene promoters

Supplementary Data 6: Variants used in fine-mapping of 107 known T2D signals

Supplementary Data 7: Candidate causal variants in islet enhancers at known T2D signals

Supplementary Data 8: Putative T2D variants genome-wide in islet enhancers

Supplementary Data 9: Topologically associating domains in pancreatic islets
