# Supplementary material for: Pancreatic islet chromatin accessibility and conformation reveals distal enhancer networks of type 2 diabetes risk
Source: Nat Commun. 2019 May 7;10:2078. doi: 10.1038/s41467-019-09975-4 (PMC6505525; doi:10.1038/s41467-019-09975-4)
Supplement: Supplementary file 3 — Supplementary Information [file 41467_2019_9975_MOESM3_ESM.pdf]

Pancreatic islet chromatin accessibility and conformation reveals distal enhancer networks of type 2 diabetes risk

Greenwald et al.

<u>**Supplementary Figures**</u>**:**

Supplementary Figure 1: Characteristics of pancreatic islet accessible chromatin sites

Supplementary Figure 2: Characteristics of pancreatic islet chromatin loops

Supplementary Figure 3: Effects of variants in pancreatic islet accessible chromatin on T2D risk

Supplementary Figure 4: T2D enhancer signal chromatin loops to candidate target genes

Supplementary Figure 5: Candidate causal islet enhancer variant at *IGF2BP2*

Supplementary Figure 6: Characterization of mice after conditional *Imp2/IGF2BP2* ablation in beta cells

<u>**Supplementary Tables**</u>**:**

Supplementary Table 1: Donor and sequencing characteristics of pancreatic islet samples

Supplementary Table 2: Functional annotations enriched in genes with multiple enhancer loops

Supplementary Table 3: Candidate target genes of T2D islet enhancer signals

Supplementary Table 4: Target genes with islet eQTLs for T2D islet enhancer signals

Supplementary Table 5: Gene set annotations enriched in target genes of T2D islet enhancer signals

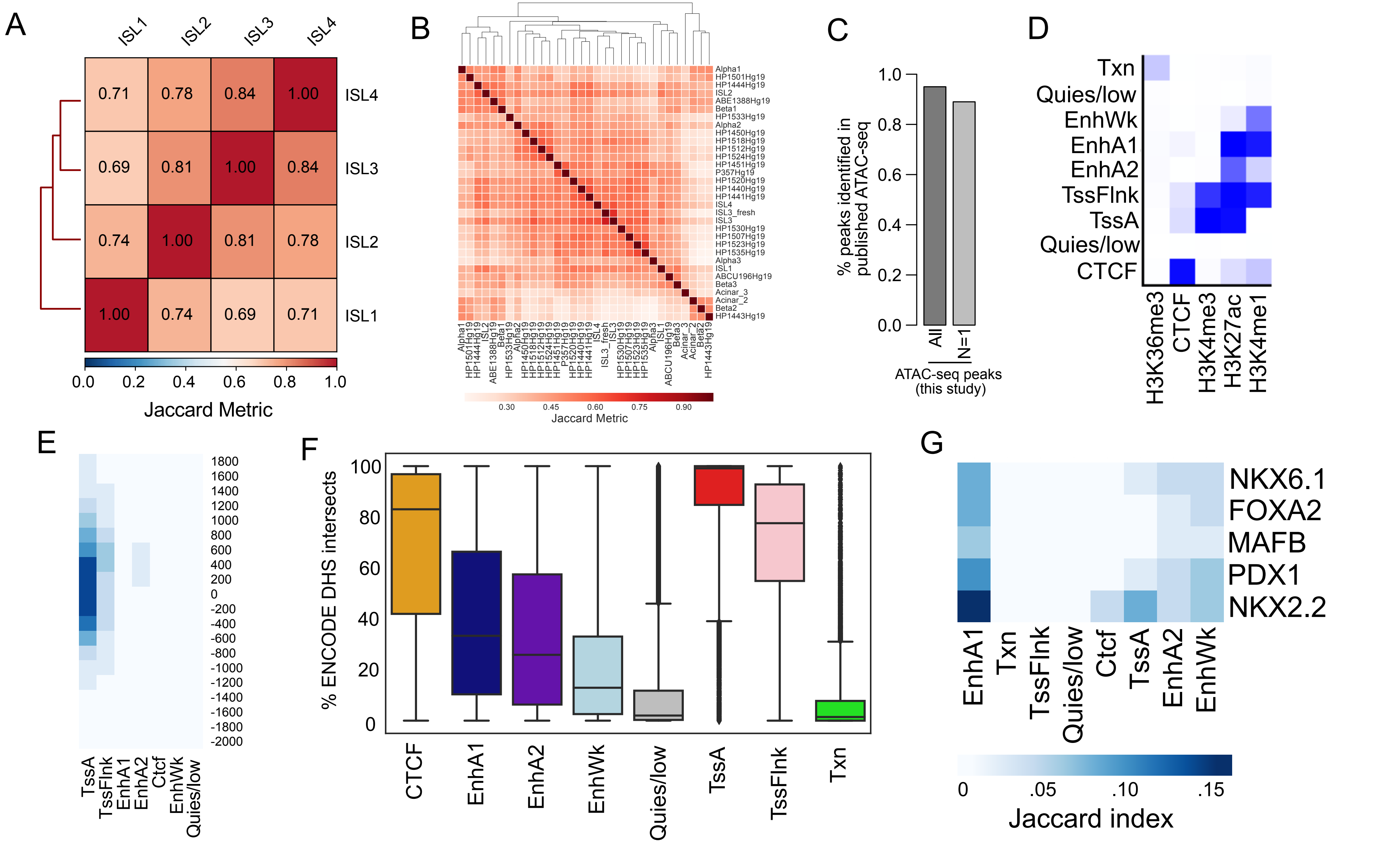

Supplementary Figure 1. Characteristics of pancreatic islet accessible chromatin sites (A) Heatmap of the Spearman correlation between ATAC-seq read coverage in merged peaks across four islet samples (ISL1-4). (B) Jaccard overlap between peak calls for the four islet samples (ISL1-4), one sample with additional data generated from fresh cells (ISL3_fresh), 19 islet samples from two published studies, and sorted alpha, beta and acinar cells from a published study. (C) Percentage of ATAC-seq sites identified across all four samples from this project (All) or just in one sample (N=1) that are also identified in sites from 19 published studies. (D) Heatmap of emission matrix probabilities for the 9-state islet model from chromHMM, with individual ChIP-seq assays shown on the x-axis and labelled chromatin states on the y-axis. (E) Heatmap showing percentage of islet accessible chromatin sites in each chromatin state mapping in 200bp bins around GENCODE transcription start sites. (F) Percentage of ENCODE cell-types in DHS sites overlapping islet accessible chromatin sites in each chromatin state. (G) Jaccard overlap of islet accessible chromatin sites with islet ChIP-seq sites for five transcription factors.

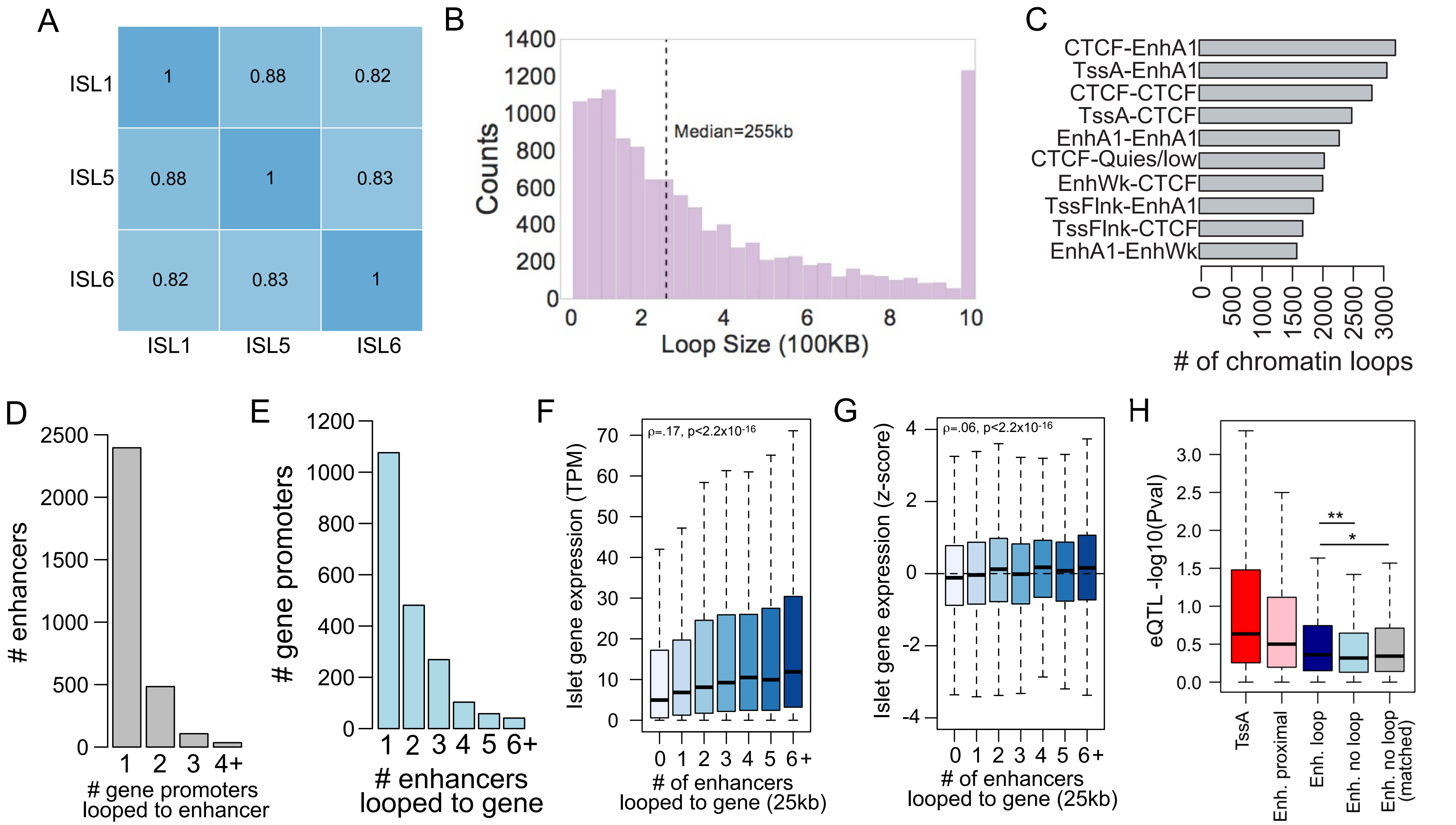

Supplementary Figure 2. Characteristics of pancreatic islet chromatin loops (A) Heatmap showing the Spearman correlation of Hi-C contacts across islet samples in 100kb bins across the genome. (B) Histogram of distance between chromatin loop anchor mid-points. (C) Number of chromatin loops containing pairs of islet accessible chromatin sites. Top 10 most frequent pairings shown. (D) Histogram of number of gene promoters directly looped to each enhancer. (E) Histogram of number of enhancers directly looped to each gene promoter. (F) Boxplot of islet expression level of genes grouped by number of enhancers in chromatin loop to gene promoter using a 25kb flanking window around loop boundaries. (G) Boxplot of relative islet gene expression level normalized across 53 GTEx tissues grouped by number of enhancers in chromatin loop to gene promoter using a 25kb flanking window around loop boundaries. (H) Expression QTL p-values of variants in active promoters (TssA; red), enhancers proximal to gene promoters (Enh. proximal; pink), enhancers in loops to gene promoters within a 25kb window around loops, (Enh. loop dark blue), enhancers not in loops to gene promoters within a 25kb window around loops including all enhancers (Enh. no-loop; light blue) and distance-matched enhancers (Enh. no-loop matched; grey). Wilcox *P<.01, **P<.00001).

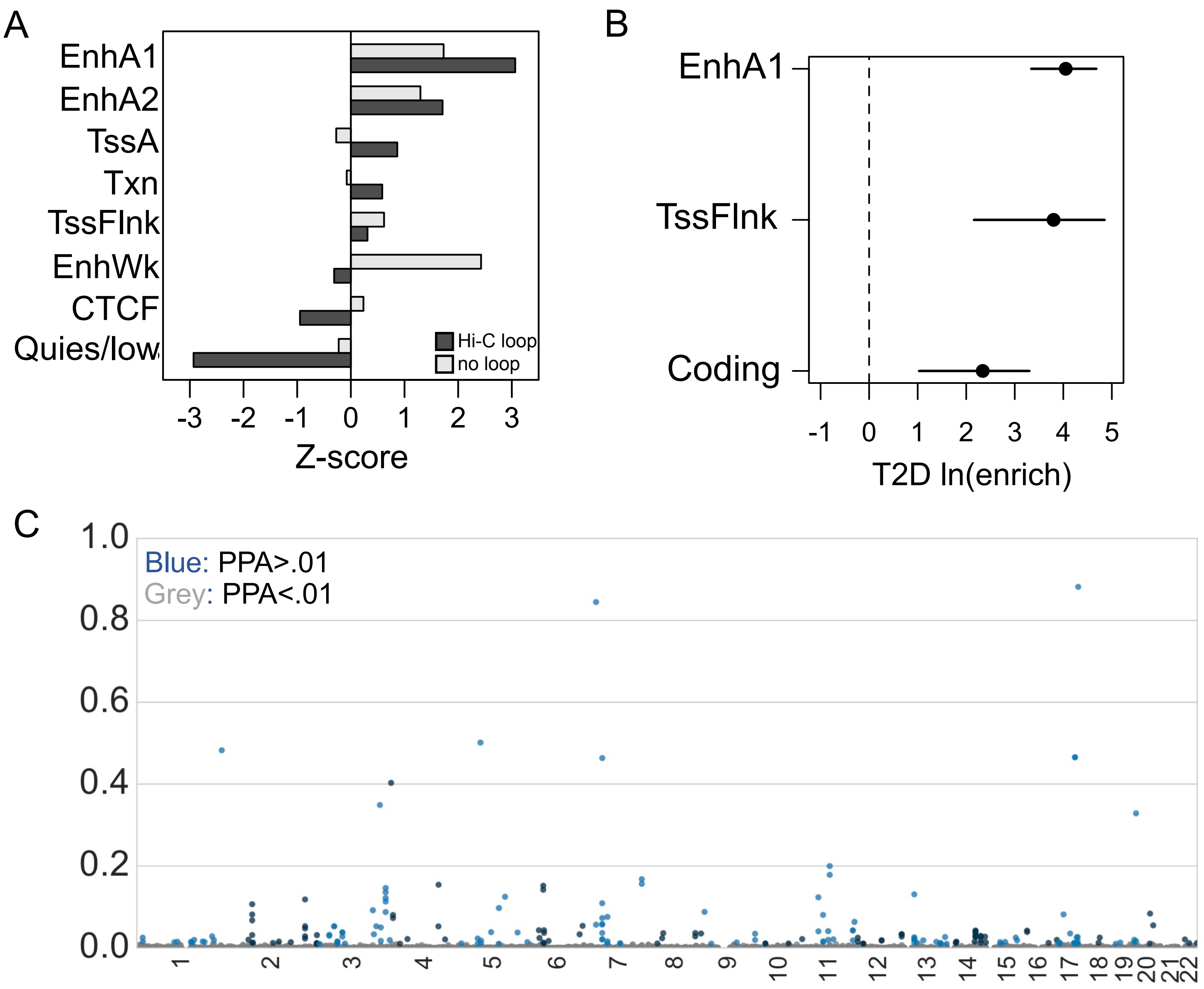

Supplementary Figure 3. Effects of variants in pancreatic islet accessible chromatin on T2D risk (A) Enrichment Z-score measured using LD-score regression for each class of islet accessible chromatin (y-axis), subset by states that were (dark) or were not (light) within 25kb of a Hi-C loop anchor. (B) Enrichments from the fgwas genome-wide joint model including islet active enhancers (EnhA1), flanking promoters (TssFlnk), and coding exons (CDS). Values represent log enrichment and 95% CI. (C) Posterior causal probabilities (PPA) of variants within islet active enhancers in 1MB windows genome-wide excluding known T2D risk loci. Blue = PPA>0.01, grey = PPA<0.01.

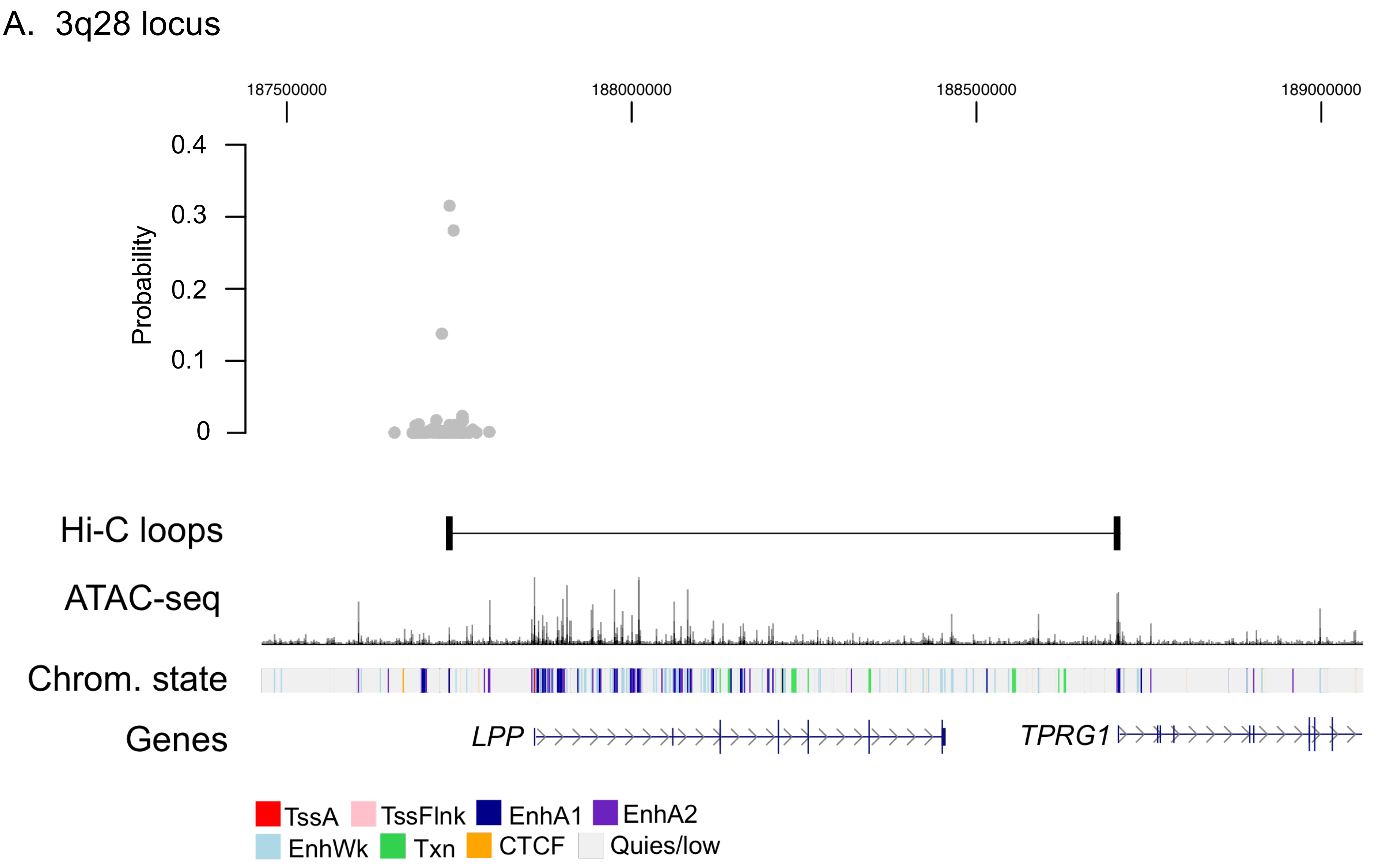

Supplementary Figure 4. T2D enhancer signal chromatin loops to candidate target genes. Re-weighted posterior causal probabilities of variants (top), islet Hi-C loops, chromatin states and ATAC-seq signal (middle), and known genes (bottom), for T2D signals at (A) the 3q28 locus, (B) the 10p13 locus, (C) the 11p15 locus, and (D) the 10q22 locus. For (C), posterior probabilities are shown in different colors for each of the five independent signals at this locus.

B. 10p13 locus

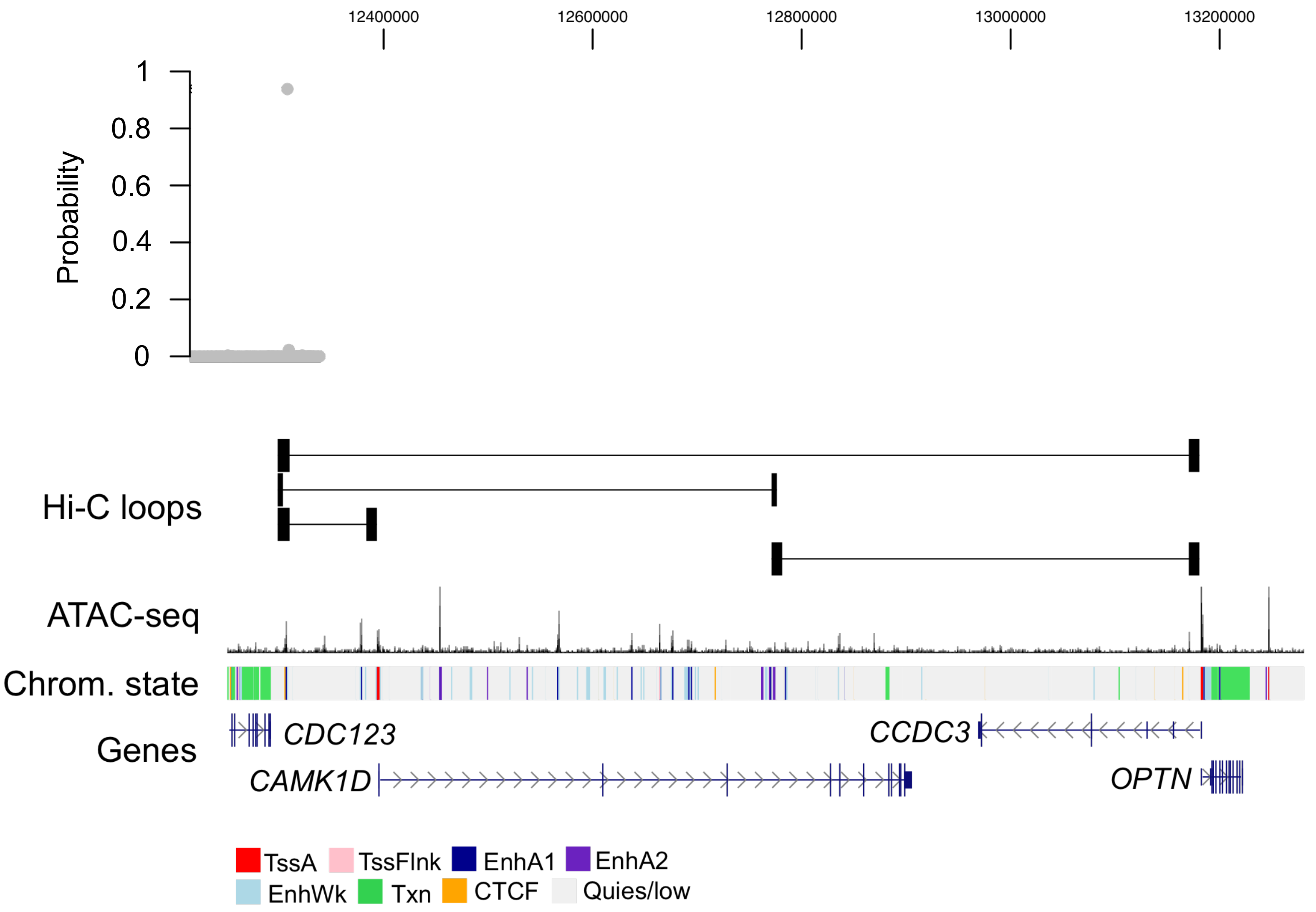

C. 11p15 locus

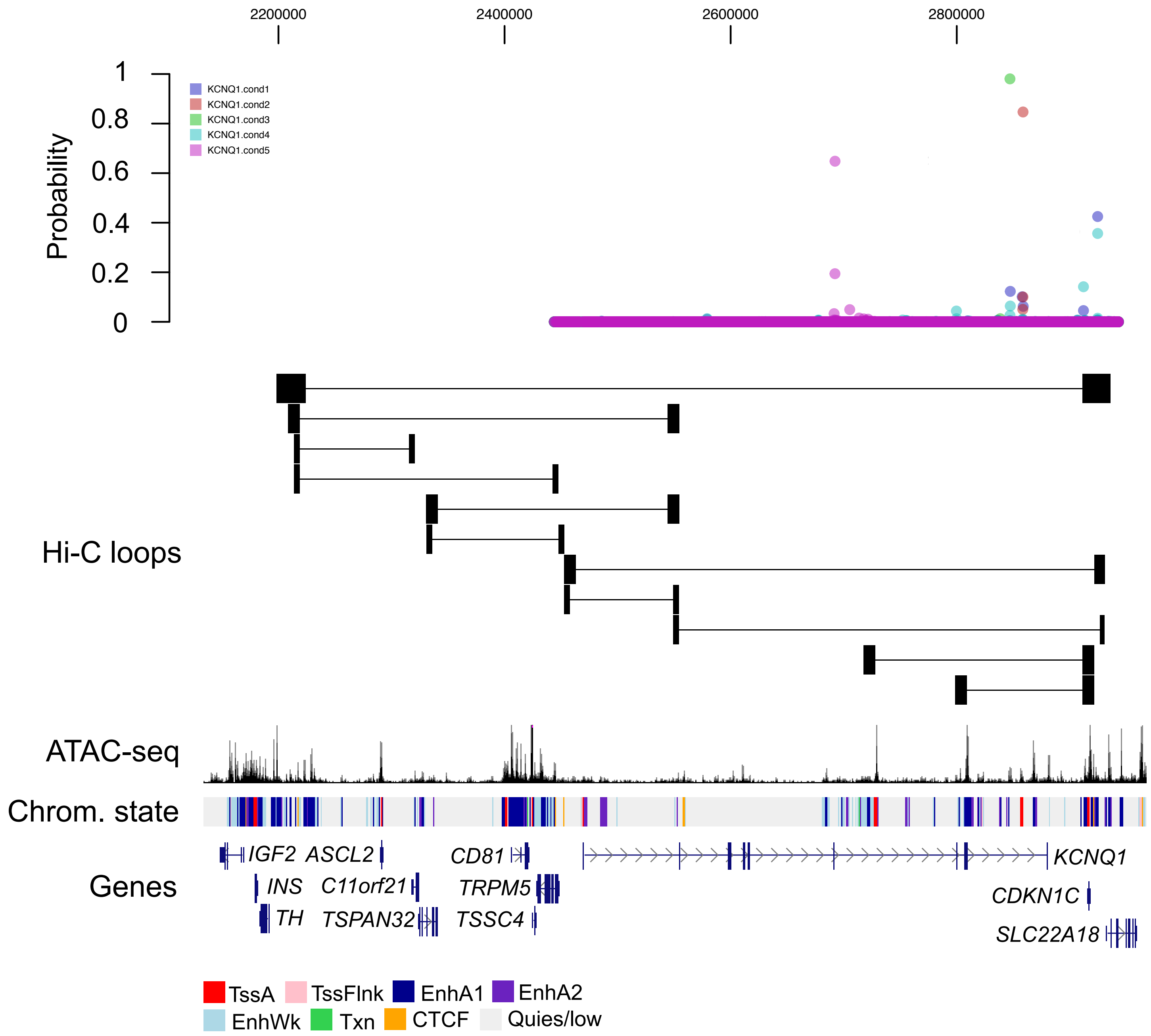

D. 10q22 locus

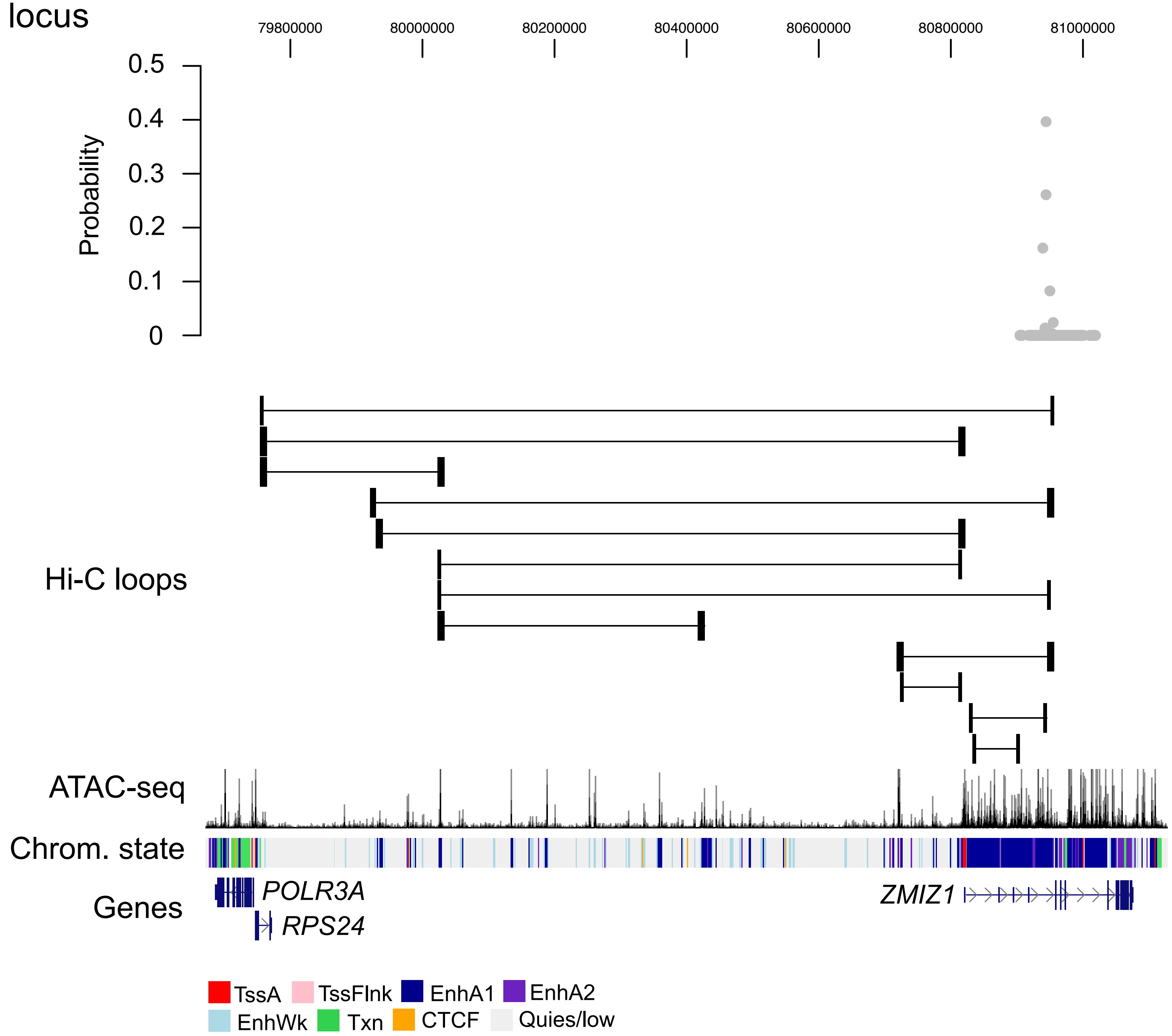

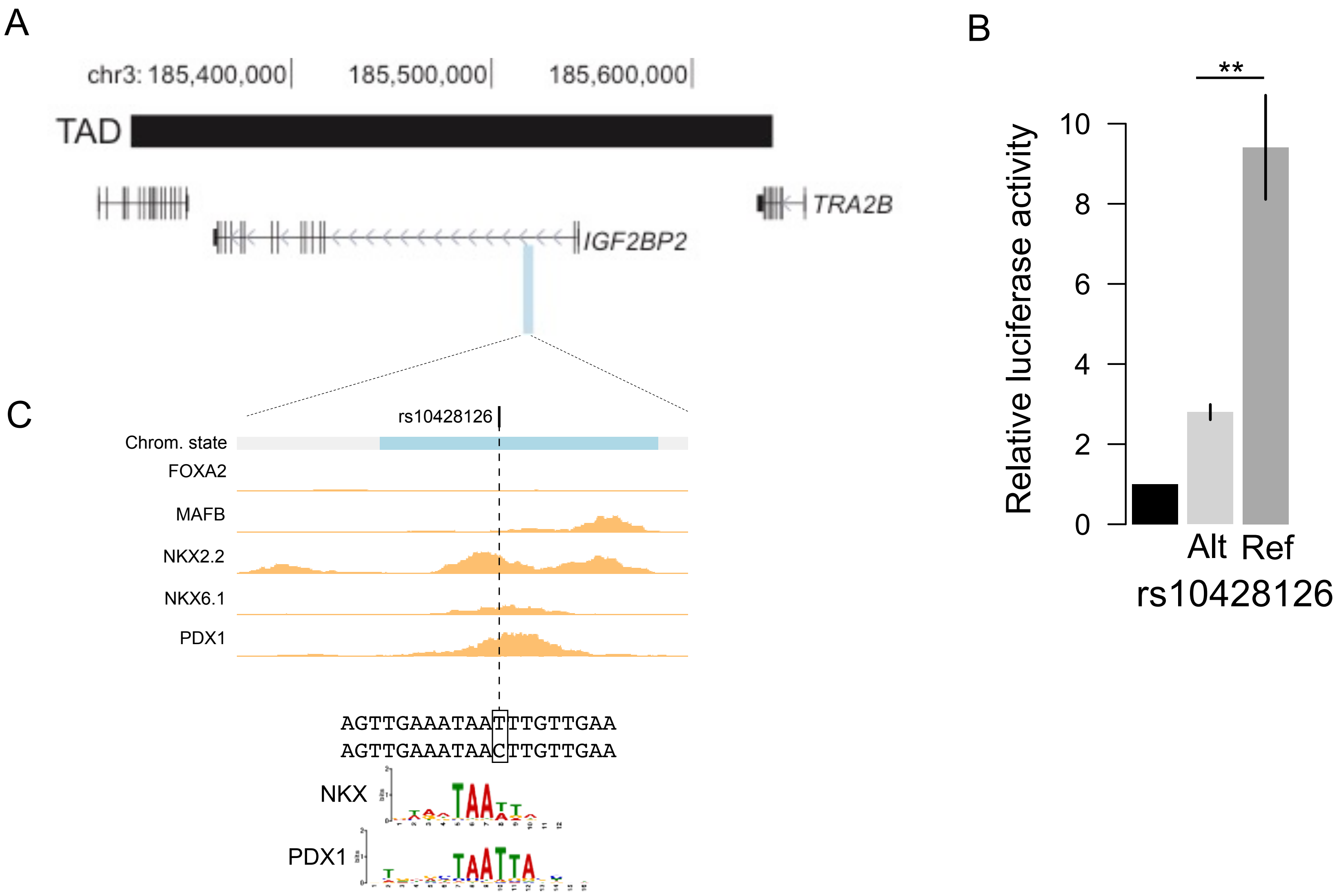

Supplementary Figure 5. Candidate causal islet enhancer variant at *IGF2BP2*. (A) Genomic region showing location of topologically-associating domain (TAD) containing T2D risk variants at the *IGF2BP2* locus. *IGF2BP2* is the only gene promoter that maps in the TAD. Light blue bar represents the region containing T2D risk variants and which is proximal (within 25kb) to the *IGF2BP2* promoter. (B) T2D variant 10428126 has allelic effects on islet enhancer activity in the islet cell line MIN6 where the T2D risk and alternate (alt) allele C has reduced activity than the reference (ref) allele (T-test P=.001; N=3). (C) T2D variant rs10428126 maps in an enhancer element bound by PDX1 and NKX2.2 in primary islets, and the risk allele is predicted to disrupt NKX and PDX1 binding motifs among other factors.

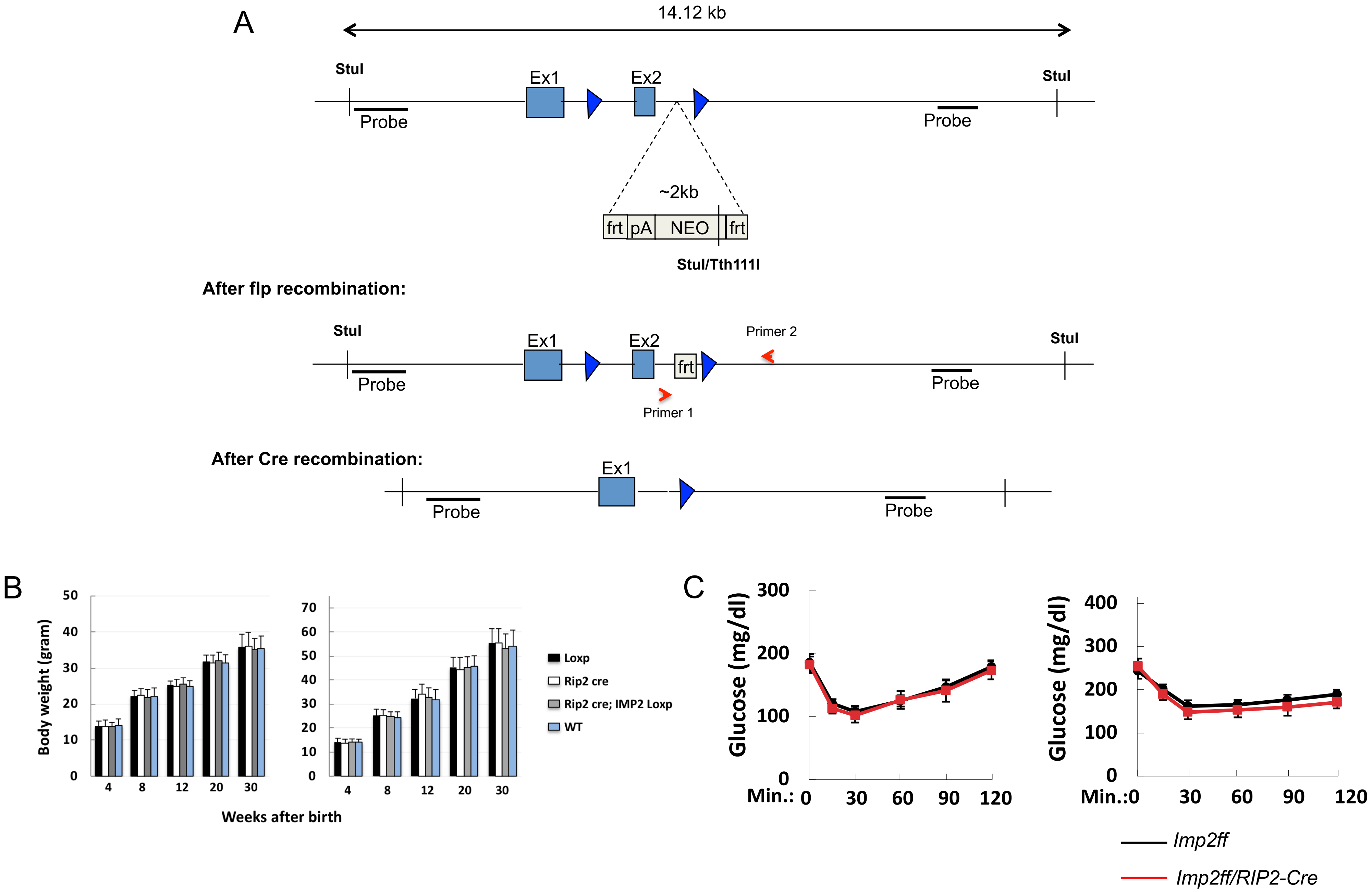

Supplementary Figure 6. Characterization of mice after conditional *Imp2/IGF2BP2* ablation in beta cells. (A) Schematic representation of the WT *Imp2* allele showing exon 1-2 and flanking intron sequences, and the $Imp2^{flox}$ targeted allele. (B) Body weight for WT, *RIP2-Cre*, $Imp2^{ff}$ and $Imp2^{ff}$*/RIP2-Cre* mice on normal chow diet (left) and high fat diet (right). (C) Insulin tolerance tests in 14-week-old $Imp2^{ff}$*/RIP2-Cre* (red) and $Imp2^{ff}$ (black) mice on a normal chow (left) and high fat diet (right). Values are mean and SD.

Supplementary Table 1. Donor and sequencing characteristics of pancreatic islet samples

Subject ID			Donor Information											Sequencing Information								
Paper Sample ID	Sample Source	UNOS ID	Ancestry	Sex	Age	BMI	HbA1c	Diabetes Status	Medical History	Medication History	Cause of Death	Islet Viability %[x]	Islet Purity %[x]	ATAC-seq performed?	ATAC-seq read depth[*]	Mitochondrial read %	Fraction of unique reads in peaks (FRiP)	TSS enrichment (ENCODE metric)	ATAC-seq peaks (overlap, if applicable)	Hi-C performed?	Hi-C read depth[+]	Hi-C loops
ISL1	IIDP/Univ. of Miami	ACLI344A	Caucasian	Female	59	24.7	5.4	Non-diabetic	N/A	N/A	CNS tumor	95.0	90.0	Yes (frozen)	27,325,062	37.98	0.34	5.06	61,053	Yes	1,794,763,577	2,393
ISL2	IIDP/Univ. of Pennsylvania	ACLM016A	Caucasian	Male	55	23.2	5.2	Non-diabetic	N/A	N/A	Head trauma	93.0	90.0	Yes (frozen)	17,225,636	59.88	0.32	6.08	45,453	No	-	-
														Yes (frozen)	68,604,176	31.94	0.46	6.50	76,833 (74,831)			
ISL3	IIDP/Scharp-Lacy Institute	AEEM022	East Asian	Male	57	29.1	5.3	Non-diabetic	Clean	N/A	Stroke	95.0	90.0	Yes (fresh)	71,568,982	28.08	0.71	7.80	96,779 (74,831)	No	-	-
ISL4	IIDP/Scharp-Lacy Institute	AEFB055	Caucasian	Male	35	31.5	4.9	Non-diabetic	Knee surgery	N/A	Head trauma	95.0	90.0	Yes (frozen)	73,136,422	31.54	0.28	4.36	70,278	No	-	-
ISL5	IIDP/Scharp-Lacy Institute	ADAM295	African American	Female	56	33.4	5.2	Non-diabetic	Hypertension	N/A	Stroke	95.0	90.0	No	-	-	-	-	-	Yes	1,558,203,805	9,910
ISL6	IIDP/City of Hope	AEDU239	Caucasian	Male	53	29.0	5.5	Non-diabetic	Hypertension, Multifocal cellular carinoma of the kidneys	N/A	Stroke	N/A	78.0	No	-	-	-	-	-	Yes	538,807,603	4,571

[x] Viability and purity before further purification by hand-picking islets
[*] After alignment, filtering for autosomal reads, removing duplicates and low mapping quality reads. Total reads counting both read1 and read2.
[+] After alignment, total reads counting both read1 and read2.

Supplementary Table 2. Functional annotations enriched in genes with multiple enhancer loops

Gene Set Name*	# Genes in Gene Set	# Genes in Overlap	p-value	FDR q-value
Biological Process				
GO_REGULATION_OF_TRANSCRIPTION_FROM_RNA_POLYMERASE_II_PROMOTER	1784	132	2.44E-44	1.08E-40
GO_REGULATION_OF_CELL_DIFFERENTIATION	1492	113	8.88E-39	1.74E-35
GO_POSITIVE_REGULATION_OF_MOLECULAR_FUNCTION	1791	124	1.18E-38	1.74E-35
GO_POSITIVE_REGULATION_OF_BIOSYNTHETIC_PROCESS	1805	123	1.19E-37	1.32E-34
GO_POSITIVE_REGULATION_OF_RESPONSE_TO_STIMULUS	1929	126	9.72E-37	8.63E-34
GO_POSITIVE_REGULATION_OF_GENE_EXPRESSION	1733	117	2.10E-35	1.55E-32
GO_REGULATION_OF_MULTICELLULAR_ORGANISMAL_DEVELOPMENT	1672	114	7.10E-35	4.50E-32
GO_NEGATIVE_REGULATION_OF_GENE_EXPRESSION	1493	106	6.51E-34	3.61E-31
GO_RESPONSE_TO_ENDOGENOUS_STIMULUS	1450	102	2.68E-32	1.32E-29
GO_REGULATION_OF_INTRACELLULAR_SIGNAL_TRANSDUCTION	1656	109	5.29E-32	2.10E-29
GO_INTRACELLULAR_SIGNAL_TRANSDUCTION	1572	106	5.30E-32	2.10E-29
GO_POSITIVE_REGULATION_OF_CATALYTIC_ACTIVITY	1518	104	5.69E-32	2.10E-29
GO_POSITIVE_REGULATION_OF_CELL_COMMUNICATION	1532	104	1.22E-31	4.17E-29
GO_CELLULAR_RESPONSE_TO_ORGANIC_SUBSTANCE	1848	115	1.40E-31	4.43E-29
GO_NEGATIVE_REGULATION_OF_NITROGEN_COMPOUND_METABOLIC_PROCESS	1517	103	2.43E-31	7.17E-29
GO_NEUROGENESIS	1402	98	8.12E-31	2.25E-28
GO_REGULATION_OF_CELL_PROLIFERATION	1496	101	1.55E-30	4.05E-28
GO_POSITIVE_REGULATION_OF_PROTEIN_METABOLIC_PROCESS	1492	100	5.54E-30	1.37E-27
GO_NEGATIVE_REGULATION_OF_TRANSCRIPTION_FROM_RNA_POLYMERASE_II_PROMOTER	740	70	6.68E-30	1.56E-27
GO_RESPONSE_TO_OXYGEN_CONTAINING_COMPOUND	1381	95	2.38E-29	5.29E-27
GO_REGULATION_OF_PROTEIN_MODIFICATION_PROCESS	1710	106	5.86E-29	1.24E-26
GO_CELL_DEVELOPMENT	1426	95	2.70E-28	5.44E-26
GO_NEGATIVE_REGULATION_OF_RESPONSE_TO_STIMULUS	1360	92	6.74E-28	1.30E-25
GO_REGULATION_OF_PHOSPHORUS_METABOLIC_PROCESS	1618	101	7.81E-28	1.44E-25
GO_NEGATIVE_REGULATION_OF_CELL_COMMUNICATION	1192	85	2.27E-27	4.03E-25
GO_TRANSCRIPTION_FROM_RNA_POLYMERASE_II_PROMOTER	724	66	2.57E-27	4.38E-25
GO_REGULATION_OF_TRANSPORT	1804	105	1.71E-26	2.81E-24
GO_POSITIVE_REGULATION_OF_TRANSCRIPTION_FROM_RNA_POLYMERASE_II_PROMOTER	1004	76	3.80E-26	6.02E-24
GO_PHOSPHATE_CONTAINING_COMPOUND_METABOLIC_PROCESS	1977	109	1.38E-25	2.11E-23
GO_CELLULAR_RESPONSE_TO_STRESS	1565	95	2.61E-25	3.87E-23
Cellular component				
GO_CYTOSKELETON	1967	103	1.64E-22	9.53E-20
GO_NEURON_PART	1265	75	1.55E-19	3.50E-17
GO_CELL_JUNCTION	1151	71	1.81E-19	3.50E-17
GO_CELL_PROJECTION	1786	89	3.64E-18	5.27E-16
GO_NUCLEOPLASM_PART	708	50	2.46E-16	2.85E-14
GO_CATALYTIC_COMPLEX	1038	61	6.66E-16	6.44E-14
GO_INTRINSIC_COMPONENT_OF_PLASMA_MEMBRANE	1649	80	9.12E-16	7.56E-14
GO_CYTOSKELETAL_PART	1436	73	1.70E-15	1.14E-13
GO_NEURON_PROJECTION	942	57	1.77E-15	1.14E-13
GO_GOLGI_APPARATUS	1445	73	2.34E-15	1.36E-13
GO_SYNAPSE	754	50	2.96E-15	1.56E-13
GO_INTRACELLULAR_VESICLE	1259	65	3.22E-14	1.50E-12
GO_SYNAPSE_PART	610	43	3.37E-14	1.50E-12
GO_TRANSFERASE_COMPLEX	703	46	6.30E-14	2.61E-12
GO_MEMBRANE_REGION	1134	58	1.20E-12	4.66E-11
GO_MITOCHONDRION	1633	72	2.67E-12	9.68E-11
GO_ANCHORING_JUNCTION	489	35	5.42E-12	1.85E-10
GO_MICROTUBULE_CYTOSKELETON	1068	54	1.14E-11	3.62E-10
GO_NUCLEOLUS	848	47	1.18E-11	3.62E-10
GO_VACUOLE	1180	57	1.79E-11	5.19E-10
GO_PLASMA_MEMBRANE_REGION	929	48	7.90E-11	2.18E-09
GO_NUCLEAR_BODY	349	27	2.62E-10	6.90E-09
GO_MICROTUBULE_ORGANIZING_CENTER	623	37	2.83E-10	7.14E-09
GO_CELL_SUBSTRATE_JUNCTION	398	28	1.06E-09	2.55E-08
GO_CELL_PROJECTION_PART	946	46	1.36E-09	3.15E-08
GO_ENDOPLASMIC_RETICULUM	1631	65	1.95E-09	4.35E-08
GO_GOLGI_APPARATUS_PART	893	44	2.09E-09	4.49E-08
GO_TRANSCRIPTION_FACTOR_COMPLEX	298	23	5.73E-09	1.19E-07
GO_POSTSYNAPSE	378	26	6.61E-09	1.32E-07
GO_CYTOPLASMIC_SIDE_OF_MEMBRANE	170	17	1.26E-08	2.40E-07
Pathway				
KEGG_PATHWAYS_IN_CANCER	328	31	6.49E-14	1.21E-11
KEGG_INSULIN_SIGNALING_PATHWAY	137	16	3.58E-09	3.33E-07
KEGG_WNT_SIGNALING_PATHWAY	151	15	9.80E-08	6.08E-06
KEGG_MELANOGENESIS	102	11	2.20E-06	8.81E-05
KEGG_PRION_DISEASES	35	7	2.42E-06	8.81E-05
KEGG_CELL_CYCLE	128	12	3.41E-06	8.81E-05
KEGG_P53_SIGNALING_PATHWAY	69	9	3.79E-06	8.81E-05
KEGG_FOCAL_ADHESION	201	15	3.79E-06	8.81E-05
KEGG_ENDOCYTOSIS	183	14	5.94E-06	1.23E-04
KEGG_NOTCH_SIGNALING_PATHWAY	47	7	1.88E-05	3.43E-04
KEGG_GLIOMA	65	8	2.03E-05	3.43E-04
KEGG_MAPK_SIGNALING_PATHWAY	267	16	2.88E-05	4.46E-04
KEGG_MELANOMA	71	8	3.89E-05	5.30E-04
KEGG_UBIQUITIN_MEDIATED_PROTEOLYSIS	138	11	3.99E-05	5.30E-04
KEGG_ADHERENS_JUNCTION	75	8	5.79E-05	7.18E-04
KEGG_SMALL_CELL_LUNG_CANCER	84	8	1.30E-04	1.51E-03
KEGG_TGF_BETA_SIGNALING_PATHWAY	86	8	1.53E-04	1.66E-03
KEGG_N_GLYCAN_BIOSYNTHESIS	46	6	1.61E-04	1.66E-03
KEGG_PROSTATE_CANCER	89	8	1.95E-04	1.91E-03
KEGG_GAP_JUNCTION	90	8	2.11E-04	1.96E-03
KEGG_ALZHEIMERS_DISEASE	169	11	2.44E-04	2.16E-03
KEGG_CHRONIC_MYELOID_LEUKEMIA	73	7	3.27E-04	2.76E-03
KEGG_CALCIUM_SIGNALING_PATHWAY	178	11	3.80E-04	3.08E-03
KEGG_JAK_STAT_SIGNALING_PATHWAY	155	10	4.95E-04	3.83E-03
KEGG_SNARE_INTERACTIONS_IN_VESICULAR_TRANSPORT	38	5	5.48E-04	4.07E-03
KEGG_ALDOSTERONE_REGULATED_SODIUM_REABSORPTION	42	5	8.75E-04	6.26E-03
KEGG_MATURITY_ONSET_DIABETES_OF_THE_YOUNG	25	4	9.40E-04	6.48E-03
KEGG_VASOPRESSIN_REGULATED_WATER_REABSORPTION	44	5	1.09E-03	7.21E-03
KEGG_TYPE_II_DIABETES_MELLITUS	47	5	1.47E-03	9.41E-03
KEGG_RENAL_CELL_CARCINOMA	70	6	1.57E-03	9.72E-03

* Top 30 gene sets shown for each category

Supplementary Table 3. Candidate target genes of T2D islet enhancer signals

T2D signal*	Candidate target genes*	# target genes
4q35 / ACSL1	ACSL1	1
3q21 / ADCY5	HACD2	1
8p11 / ANK1	NKX6-3, ANK1	2
15q26 / AP3S2	ANPEP, MESP1, MESP2	3
15q22 / C2CD4A	ICE2, RORA, C2CD4B, VPS13C, C2CD4A, RORA	6
10p13 / CDC123	**CCDC3**, **OPTN**, **CAMK1D**	3
9p21 / CDKN2B (signal 1)	-	0
9p21 / CDKN2B (signal 2)	-	0
7p22 / DGKB (signal 1)	-	0
7p13 / GCK	POLD2, DDX56, NUDCD3, GCK, TMED4, YKT6, **MYL7**, NPC1L1	8
19q13 / GIPR (signal 2)	GPR4, SYMPK, FOXA3, IRF2BP1, PPM1N, QPCTL, VASP, MYPOP, EML2, NANOS2, OPA3, SNRPD2, RTN2, GIPR	14
9p24 / GLIS3	GLIS3	1
3q27 / IGF2BP2	IGF2BP2	1
11p15.1 / KCNJ11	ABCC8, KCNJ11	2
11p15 / KCNQ1 (signal 1)	TRPM5, KCNQ1, TSSC4, SLC22A18, PHLDA2, **CDKN1C**, **SLC22A18AS**, TH, INS, INS-IGF2	10
11p15 / KCNQ1 (signal 2)	-	
11p15 / KCNQ1 (signal 4)	TRPM5, KCNQ1, TSSC4, SLC22A18, PHLDA2, **CDKN1C**, **SLC22A18AS**, TH, INS, INS-IGF2	10
12p11 / KLHDC5	MRPS35, MANSC4	2
3q28 / LPP	**TPRG1**, **TPRG1-AS1**	2
12q24 / MPHOSPH9	C12orf65, PITPNM2-AS1, SNRNP35, OGFOD2, TMED2, CDK2AP1, MPHOSPH9, PITPNM2, ARL6IP4, DDX55, KNTC1, ABCB9, HIP1R	13
15q14 / RASGRP1	FAM98B, C15orf53, RASGRP1	3
6q23 / SLC35D3	NHEG1	1
17p13 / SRR	HIC1, TSR1, OVCA2, SGSM2, MNT, SMG6	6
4q31 / TMEM154	**FBXW7**, **LINC02486**	2
2q21 / TMEM163	**CCNT2**, ACMSD, TMEM163	3
8q22 / TP53INP1	**TP53INP1**, **NDUFAF6**	2
4p16 / WFS1	C4orf50, WFS1	2
5q13 / ZBED3	PDE8B	1
6p21 / ZFAND3	-	0
10q22 / ZMIZ1	LINC00595, POLR3A, RPS24, ZMIZ1	4

* Locus where signal is located, and nearby gene used to designate locus. Where a locus contains multiple signals the signal number is in parentheses

* T2D enhancer variants within 25kb of chromatin loops or promoter-proximity to the gene promoter. Genes in direct loops to T2D enhancers in **bold**

Supplementary Table 4. Candidate target genes with islet eQTL for T2D islet enhancer signals

Variant	Gene	eQTL p-value*	Shared**
Known signals			
rs11257655	*CAMK1D*	1.72E-14	Y
rs508419	*NKX6-3*	5.59E-10	Y
rs1260294	*ABCB9*	2.63E-07	Y
rs7646518	*IGF2BP2*	7.49E-07	Y
rs4954179	*ACMSD*	5.43E-06	Y
rs4954179	*TMEM163*	9.69E-05	Y
rs17205526	*C2CD4B*	0.00088	Y
rs116401167	*ACSL1*	0.040	Y
rs7732130	*PDE8B*	0.048	Y
Novel signals			
rs34584161	*RNF6*	7.65E-10	Y
rs174564	*FADS1*	6.61E-09	Y
rs2723087	*CEP68*	2.29E-08	Y
rs17854357	*SNX32*	2.89E-08	Y
rs5415	*CLDN7*	4.68E-07	Y
rs34584161	*CDK8*	1.97E-06	Y
rs1132414	*SPATA20*	1.07E-05	Y
rs2564940	*ALAS1*	3.65E-05	Y
rs10132336	*SYNDIG1L*	4.76E-05	Y
rs59630333	*SCRN2*	0.00025	Y
rs7151785	*STXBP6*	0.00047	Y
rs1317840	*VEGFA*	0.0045	Y

* Genes with eQTL p-values<.05 after correction for target genes tested at a given signal

** Bayesian co-localization probability of a shared causal variant greater than two distinct causal variants

Supplementary Table 5. Gene set annotations enriched in target genes of T2D islet enhancer signals

Target genes

Gene Set Name	# Genes in Gene Set	# Genes in Overlap	p-value	FDR q-value*
Biological Process				
GO_REGULATION_OF_TRANSPORT	1804	17	1.37E-08	3.42E-05
GO_NEGATIVE_REGULATION_OF_CELL_COMMUNICAT	1192	14	2.09E-08	3.42E-05
GO_CARBOHYDRATE_HOMEOSTASIS	170	7	2.31E-08	3.42E-05
GO_REGULATION_OF_SECRETION	699	10	4.42E-07	4.91E-04
GO_HOMEOSTATIC_PROCESS	1337	13	6.05E-07	5.37E-04
GO_POSITIVE_REGULATION_OF_TRANSPORT	936	11	7.81E-07	5.78E-04
GO_ESTABLISHMENT_OF_LOCALIZATION_IN_CELL	1676	14	1.29E-06	8.03E-04
GO_NEGATIVE_REGULATION_OF_SECRETION	200	6	1.56E-06	8.03E-04
GO_GOLGI_VESICLE_TRANSPORT	319	7	1.63E-06	8.03E-04
GO_REGULATION_OF_PEPTIDE_SECRETION	209	6	2.01E-06	8.92E-04
Cellular component				
GO_VESICLE_MEMBRANE	512	9	3.16E-07	1.83E-04
GO_ENDOPLASMIC_RETICULUM	1631	14	9.37E-07	2.30E-04
GO_CYTOPLASMIC_VESICLE_PART	601	9	1.19E-06	2.30E-04
GO_INTRACELLULAR_VESICLE	1259	12	2.10E-06	3.05E-04
GO_TRANSPORT_VESICLE	338	6	3.09E-05	3.58E-03
GO_ENDOPLASMIC_RETICULUM_GOLGI_INTERMEDIAT	105	4	3.74E-05	3.58E-03
GO_VACUOLE	1180	10	4.32E-05	3.58E-03
GO_SARCOLEMMA	125	4	7.38E-05	5.35E-03
GO_SECRETORY_VESICLE	461	6	1.70E-04	1.09E-02
GO_ENDOPLASMIC_RETICULUM_GOLGI_INTERMEDIAT	63	3	1.94E-04	1.13E-02
Pathway				
PID_HNF3B_PATHWAY	45	5	1.73E-08	2.31E-05
KEGG_TYPE_II_DIABETES_MELLITUS	47	4	1.51E-06	1.00E-03
KEGG_MATURITY_ONSET_DIABETES_OF_THE_YOUNG	25	3	1.18E-05	3.92E-03
REACTOME_REGULATION_OF_BETA_CELL_DEVELOPMI	30	3	2.07E-05	5.50E-03
REACTOME_REGULATION_OF_INSULIN_SECRETION	93	3	6.11E-04	1.18E-01
REACTOME_DEVELOPMENTAL_BIOLOGY	396	5	6.94E-04	1.18E-01
REACTOME_POTASSIUM_CHANNELS	98	3	7.11E-04	1.18E-01
REACTOME_INTEGRATION_OF_ENERGY_METABOLISM	120	3	1.28E-03	1.83E-01
REACTOME_INWARDLY_RECTIFYING_K_CHANNELS	31	2	1.38E-03	1.83E-01

* Gene sets with q-value <.20 listed, only top 10 sets listed for each category

** Gene set enrichment for non-target genes after removing a cluster of 13 interferon alpha genes

Islet cis eQTL target genes

Gene Set Name	# Genes in Gene Set	# Genes in Overlap	p-value	FDR q-value*
Biological Process				
GO_REGULATION_OF_VESICLE_MEDIATED_TRANSPOI	462	4	4.30E-05	4.77E-02
GO_VESICLE_MEDIATED_TRANSPORT	1239	5	1.56E-04	1.39E-01

Non-target genes

Gene Set Name**	# Genes in Gene Set	# Genes in Overlap	p-value	FDR q-value*
Biological Process				
GO_PROTEIN_LOCALIZATION	1805	57	4.46E-19	1.98E-15
GO_ESTABLISHMENT_OF_PROTEIN_LOCALIZATION	1423	50	1.12E-18	2.49E-15
GO_REGULATION_OF_PHOSPHORUS_METABOLIC_PR	1618	51	4.34E-17	5.90E-14
GO_CELLULAR_RESPONSE_TO_STRESS	1565	50	5.32E-17	5.90E-14
GO_REGULATION_OF_RESPONSE_TO_STRESS	1468	46	2.17E-15	1.93E-12
GO_REGULATION_OF_HYDROLASE_ACTIVITY	1327	43	6.10E-15	3.93E-12
GO_POSITIVE_REGULATION_OF_CELLULAR_COMPON	1152	40	6.21E-15	3.93E-12
GO_REGULATION_OF_PROTEIN_SERINE_THREONINE_	470	25	1.00E-13	5.57E-11
GO_REGULATION_OF_PROTEIN_MODIFICATION_PRO	1710	47	1.26E-13	6.23E-11
GO_REGULATION_OF_TRANSPORT	1804	48	2.20E-13	9.03E-11
Cellular Component				
GO_CYTOSKELETON	1967	55	3.86E-16	2.24E-13
GO_CYTOSKELETAL_PART	1436	44	2.03E-14	5.90E-12
GO_MICROTUBULE_CYTOSKELETON	1068	37	7.37E-14	1.42E-11
GO_EXTRACELLULAR_SPACE	1376	39	7.31E-12	9.57E-10
GO_CELL_PROJECTION	1786	45	8.25E-12	9.57E-10
GO_HIGH_DENSITY_LIPOPROTEIN_PARTICLE	26	7	1.03E-09	9.93E-08
GO_PROTEIN_LIPID_COMPLEX	40	7	2.65E-08	2.19E-06
GO_INTRACELLULAR_VESICLE	1259	30	1.02E-07	7.41E-06
GO_MICROTUBULE_ORGANIZING_CENTER	623	20	1.59E-07	1.03E-05
GO_CELL_PROJECTION_PART	946	25	1.86E-07	1.08E-05
Pathway				
REACTOME_HEMOSTASIS	466	20	1.32E-09	1.75E-06
REACTOME_PLATELET_ACTIVATION_SIGNALING_AND	208	13	1.36E-08	9.06E-06
REACTOME_GPCR_DOWNSTREAM_SIGNALING	805	23	1.50E-07	6.63E-05
REACTOME_SIGNALING_BY_GPCR	920	24	4.13E-07	1.37E-04
REACTOME_FORMATION_OF_INCISION_COMPLEX_IN	23	5	8.83E-07	2.35E-04
REACTOME_IMMUNE_SYSTEM	933	23	1.87E-06	4.15E-04
REACTOME_G_ALPHA_Q_SIGNALLING_EVENTS	184	10	2.31E-06	4.39E-04
NABA_MATRISOME	1028	24	2.84E-06	4.71E-04
REACTOME_GASTRIN_CREB_SIGNALLING_PATHWAY_	205	10	6.04E-06	8.92E-04
REACTOME_GLOBAL_GENOMIC_NER_GG_NER	35	5	7.89E-06	1.05E-03
